# The Spanish version of Face-Name Associative Memory Exam (S-FNAME) performance is related to amyloid burden in Subjective Cognitive Decline

**DOI:** 10.1038/s41598-018-21644-y

**Published:** 2018-02-28

**Authors:** Angela Sanabria, Montserrat Alegret, Octavio Rodriguez-Gomez, Sergi Valero, Oscar Sotolongo-Grau, Gemma Monté-Rubio, Carla Abdelnour, Ana Espinosa, Gemma Ortega, Alba Perez-Cordon, Anna Gailhajanet, Isabel Hernandez, Maitee Rosende-Roca, Liliana Vargas, Ana Mauleon, Domingo Sanchez, Elvira Martin, Dorene M. Rentz, Francisco Lomeña, Agustin Ruiz, Lluis Tarraga, Merce Boada, The FACEHBI study group, N. Aguilera, M. Berthier, M. Buendia, S. Bullich, F. Campos, P. Cañabate, C. Cuevas, I. de Rojas, S. Diego, S. Gil, J. Giménez, R. Gismondi, M. Gómez-Chiari, M. Guitart, B. Hernández-Olasagarre, M. Ibarria, A. Lafuente, J. Martínez, M. Moreno, S. Moreno-Grau, L. Núñez, A. Orellana, A. Páez, A. Pancho, J. Pavía, E. Pelejà, V. Pérez-Grijalba, P. Pesini, S. Preckler, J. Romero, S. Ruiz, M. Santos, M. Sarasa, M. A. Tejero, M. Torres, A. Vivas

**Affiliations:** 1 Alzheimer Research Center and Memory Clinic of Fundació ACE, Institut Català de Neurociències Aplicades, Barcelona, Spain; 2 Psychiatry Department, Hospital Universitari Vall d’Hebron, CIBERSAM, Universitat Autònoma de Barcelona, Barcelona, Spain; 3 Center for Alzheimer Research and Treatment, Departments of Neurology, Brigham and Women’s Hospital, Massachusetts General Hospital, Harvard Medical School, Boston, MA USA; 4 Servei de Medicina Nuclear, Hospital Clínic i Provincial, Barcelona, Spain; 5 Cognitive Neurology and Aphasia Unit (UNCA), University of Malaga, Malaga, Spain; 6 Piramal Imaging GmbH, Berlin, Germany; 7 Departament de Diagnòstic per la Imatge, Clínica Corachan, Barcelona, Spain; 8 Grifols®, Barcelona, Spain; 9 Araclon Biotech®, Zaragoza, Spain

## Abstract

The Face-Name Associative Memory Exam (FNAME) is a paired associative memory test created to detect memory deficits in individuals with preclinical Alzheimer’s disease (AD). Worse performance on FNAME in cognitively healthy individuals were found related to higher amyloid beta (Aβ) burden measured with Positron-Emission-Tomography using ^11^C-PiB (PiB-PET). We previously reported normative data of a Spanish version of FNAME (S-FNAME) in cognitively healthy Spanish-speaking subjects. The aim of the present study was to determine whether performance on S-FNAME was associated with Aβ burden in subjective cognitive decline (SCD) individuals. 200 SCD subjects received neurological and neuropsychological assessments, including the S-FNAME and the Word List task from the Wechsler-Memory-Scale-III (WMS-III). Moreover, they received an MRI and (18)F-Florbetaben Positron-Emission-Tomography (FBB-PET) to measure Aβ burden. Three cognitive factor composites were derived for the episodic memory measures (face-name [SFN-N], face-occupation [SFN-O] and WMS-III) to determine whether episodic memory performance was related to Aβ deposition. Higher global Aβ deposition was significantly related to worse performance on SFN-N but not with SFN-O or WMS-III Composite. Moreover, worse SFN-N performance was significantly related to higher Aβ deposition in bilateral  Posterior Cingulate Cortex. The S-FNAME may be a promising neuropsychological tool for detecting SCD individuals with preclinical AD.

## Introduction

Alzheimer’s disease (AD) is a major health problem with an estimated 50 million people worldwide living with dementia^1,2^ with 33% of clinically normal older individuals having the underlying pathology of AD. With the advent of AD prevention trials, there is a growing interest in developing new diagnostic tools to detect the first symptoms of AD that may precede mild cognitive impairment (MCI).

Subjective cognitive decline (SCD) has been proposed as a risk factor for progression to AD dementia^3,4^. It refers to the perception of memory or other cognitive problems without impairment on standardized cognitive tests. Longitudinal studies have demonstrated that cognitively healthy individuals with SCD have a greater incidence of progression to AD dementia than those who do not perceive cognitive problems^5–8^. Other cross-sectional studies have demonstrated an association between SCD and AD biomarkers in clinically normal older individuals^9–12^.

Preclinical AD, by definition, is the identification of those clinically normal individuals with AD pathological burden. However, preclinical AD cannot be diagnosed with current standard neuropsychological tools and its diagnosis relies on the positivity of the different biomarkers of AD^13^. For this reason, there is an increasing interest in developing neuropsychological tools capable of identifying the subtle cognitive deficits present in the preclinical stage of AD^14^. The Face-Name Associative Memory Exam (FNAME), developed by Rentz and colleagues^15^, is a highly demanding face-name associative memory exam, but easy to administer and found useful for detecting evidence of amyloid-β (Aβ) burden related memory impairment in cognitively healthy individuals. The authors reported that lower performance on the FNAME were related to increased Aβ deposition in frontal, precuneus, posterior cingulate and lateral parietal cortex. We recently developed and validated the Spanish version of FNAME (S-FNAME) in 110 cognitively healthy subjects^16^ and demonstrated that subjects with MCI performed worse on this complex episodic memory test than cognitively healthy subjects^17^. The main aim of the present study was to determine whether performance on S-FNAME in 200 SCD subjects was associated with Aβ burden measured with (18) F-Florbetaben Positron Emission Tomography (FBB-PET).

## Results

### Subject Characteristics

The mean age of the 200 participants was 65.81 years (range: 51–86 years). The 38% of subjects (n = 75) were men and 62% (n = 125) women. Men had a statistically significant higher level of education than women (t = 3.57, p < 0.001), but they did not differ on age (t = 0.46, p = 0.644). Demographic and clinical characteristics of the sample are detailed in Table 1.

**Table 1:** General characteristics of the participants.

	Mean	SD	Range	
Age	65.81	7.28	51	86
Education (in years)	14.87	4.68	6	28
Vocabulary WAIS-III IQ	44.00	7.36	27	61
MMSE	29.22	0.94	27	30
Global SUVR	1.22	0.15	0.95	1.98
Frontal SUVR	1.23	0.16	0.95	2.05
PPCLP SUVR	1.22	0.15	0.97	1.93

Mean age of the 200 subjects, typical deviation and range.

WAIS-III IQ: Wechsler Adult Intelligence Scale, Third edition, Intelligence Quotient; MMSE: Mini-Mental State Examination; SUVR: Standard uptake value ratio; PPCLP: Precuneus Posterior Cingulate and Lateral Parietal cortices.

### Memory Composite factor scores

We conducted a Principal Component Analysis of all the variables of the S-FNAME and the Word List Learning test from the Wechsler Memory Scale, Third Edition (WMS-III)^18^, and found that the most consistent factorial solution was constituted by a 3-factor solution, that explained 84.75% of the total variance. Factor 1 contributed to 58.22% of the total variance, grouped the S-FNAME Name scores (SFN-N Composite). Factor 2 contributed to 15.14% of the variance and was associated with the S-FNAME Occupation scores (SFN-O Composite). Factor 3 contributed to 11.39% of the variance and was associated with the Word List Learning test from the WMS-III (WMS-III Composite). The Kaiser-Meyer-Oklin value of 0.85 and Bartlett´s test of Sphericity (p < 0.001) supported the factorability of the correlation matrix. The component correlation matrix revealed that the SFN-N was significantly correlated with the WMS-III (r = 0.57, p < 0.001) and the SFN-O (r = 0.53, p < 0.001). Factorial loadings for each subject in each memory factor composite were obtained and then correlated with Aβ deposition. Table 2 shows the distribution and factorial weights of the neuropsychological variables in the three-factor-solution.

**Table 2:** Pattern and structure matrix for Principal Component Analysis with oblim rotation of 3-factor solution from which regression weighted factor scores were derived for each subject.

Pattern Coefficients				Structure Coefficients			
Tests	Component			Tests	Component		
	1	2	3		1	2	3
S-CRN30	**0.974**	0.017	0.011	S-CRN	**0.975**	−0.537	0.476
S-CRN	**0.968**	0.006	0.022	S-CRN30	**0.969**	−0.526	0.464
S-ILN	**0.945**	−0.026	−0.012	S-ILN	**0.954**	−0.545	0.445
S-CRO	−0.024	**−0.985**	−0.002	S-CRO	0.520	**−0.971**	0.363
S-CRO30	0.007	**−0.974**	−0.020	S-CRO30	0.535	**−0.969**	0.354
S-ILO	−0.005	**−0.960**	0.027	S-ILO	0.538	**−0.967**	0.391
Recognition Memory WMS-III	−0.104	0.060	**0.851**	Delayed recall WMS-III	0.510	−0.440	**0.853**
Delayed recall WMS-III	0.093	−0.093	**0.773**	Recognition Memory WMS-III	0.265	−0.207	**0.779**
Verbal Learning WMS-III	0.211	−0.080	**0.633**	Verbal Learning WMS-III	0.555	−0.438	**0.764**

S-CRN30: S-FNAME cued recall of names at 30 minutes; S-CRN: S-FNAME cued recall of names; S-ILN: S-FNAME initial learning of names; S-CRO30: S-FNAME cued recall of occupations at 30 minutes; S-CRO: S-FNAME cued recall of occupations; S-ILO: S-FNAME initial learning of occupations; WMS-III: Wechsler Memory Scale, version III.

### Relationship between Amyloid deposition and Memory Performance

**Figure 1:**
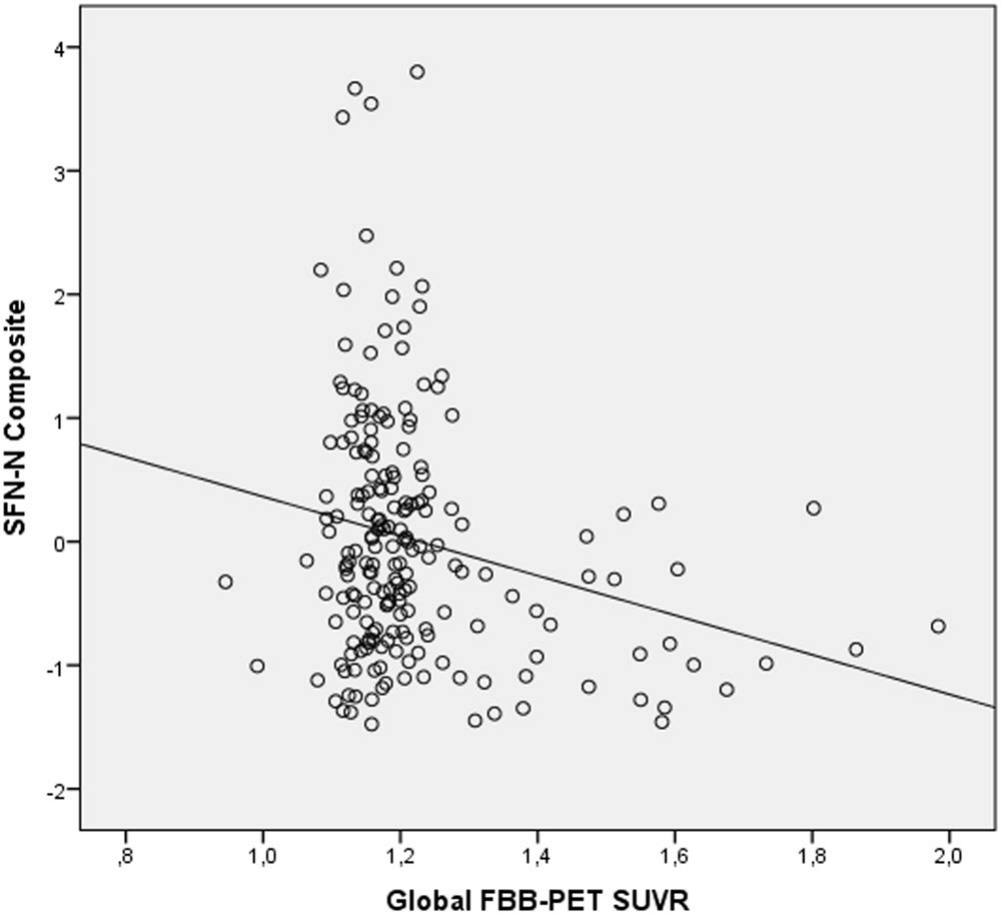
Plotting Global Amyloid against SFN-N and Global FBB-PET SUVR.

The Global Aβ score was highly correlated with Aβ deposition in Frontal (r = 0.98, p < 0.001) and Posterior Cingulate and Lateral Parietal (PPCLP) (r = 0.97, p < 0.001) cortices, thus the Global Aβ deposition score was used to explore correlations between amyloid burden and the Memory Composite factor scores. In exploring these relationships, we adjusted for age and Vocabulary of WAIS-III IQ. As detailed in Table 3, the results of multiple regression analyses showed that Global Aβ deposition was significantly negatively correlated with performance on SFN-N Composite (β = −0.22, p = 0.013) (see Fig. 1), but not significantly correlated with the SFN-O (β = −0.04, p = 0.565) or the WMS-III Composite (β = 0.01, p = 0.950). We then examined, using a Random Decision Forest analysis, whether the SFN-N was sensitive to region-specific amyloid load and found that the only statistically significant association was in the bilateral posterior cingulate cortex (PCC), where amyloid load negatively correlated with performance on this composite (β = −0.20, p = 0.01).

**Table 3:** Multiple Regression analyses of the memory factor composite scores and FBB-PET SUVR in Global cortex, corrected for age and Vocabulary WAIS-III IQ.

	Factor 1		Factor 2		Factor 3	
	S-FNAME Name (SFN-N) Composite		S-FNAME Occupation (SFN-O) Composite		Word List WMS-III (WMS-III) Composite	
ROI	β	p	β	p	β	p
Global	−0.22	0.013*	−0.04	0.565	0.01	0.950
Frontal	−0.22	0.012*	−0.07	0.413	0.01	0.958
PPCLP	−0.21	0.02*	−0.02	0.764	−0.01	0.891

S-FNAME: Spanish version of Face-Name Associative Memory Exam; ROI: Regions-of-interest; β: unstandardized partial regression coefficient. PPCLP: Precuneus, Posterior Cingulate and Lateral Parietal cortices *p < 0.05.

**Figure 2:**
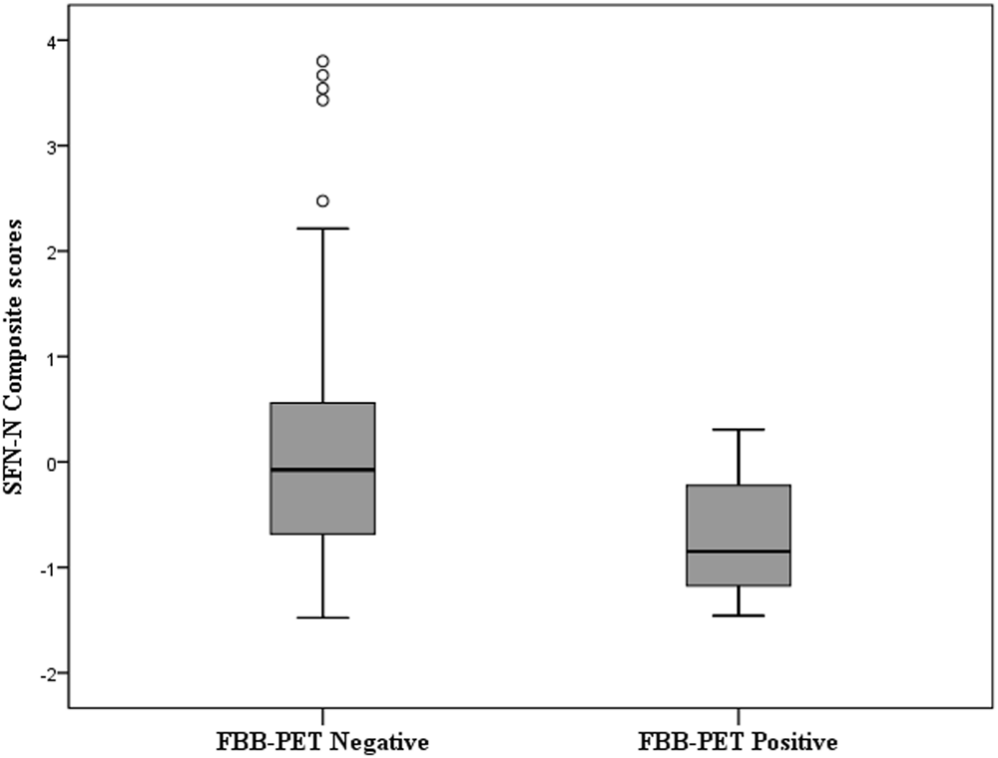
Logistic Regression revealed a significant relationship between FBB-PET positive classification and lower performance on the SFN-N Composite. A t-test comparison showed that the FBB-PET positive group performed significantly worse than the FBB-PET negative group on the SFN-N Composite.

The logistic regression analysis, adjusted for age and Vocabulary of WAIS-III IQ, showed that the global amyloid load (positive vs negative) was significantly related to SFN-N Composite (Wald = 5.75, OR = 3.13, p = 0.016), but it was not significantly related to SFN-O or WSM-III Composites. Lower scores in SFN-N Composite were associated with a higher probability to be endorsed in the positive amyloid load group. A t-test comparison showed that the global amyloid load positive group performed significantly worse than the global amyloid load negative group on the SFN-N Composite (t = 2.93, p = 0.004) (see Fig. 2), but no statistically significant differences were found between groups on SFN-O and WMS-III Composites.

To explore whether specific subtest components of the SFN-N Composite were related to Aβ amyloid deposition, we analyzed all the S-FNAME subtests of the SFN-N Composite with the Global Amyloid Deposition score. Statistically significant correlations were found after Bonferroni correction (*p < 0.016) (S-ILN: β = −0.16; p = 0.027; S-CRN: β = −0.19; p = 0.008* and S-CRN30: β = −0.19; p = 0.009*). While neither the SFN-O nor the WMS-III Composite were significantly correlated with Aβ deposition, we examined the remaining S-FNAME subtests (from SFN-O Composite) and the Word List Learning test from the WMS-III scores with the Global Amyloid Deposition score. No statistically significant correlations were found between the Global Amyloid Deposition score and performance on any of the subtests of S-FNAME Occupation (S-ILO: β = −0.06; p = 0.417; S-CRO30: β = −0.04; p = 0.540; S-CRO: β = −0.06; p = 0.412) or the Word List Learning test from the WMS-III (verbal learning: β = −0.11; p = 0.247; long-term memory: β = 0.01; p = 0.970; recognition: β = 0.01; p = 0.940).

## Discussion

The results of the present study support that S-FNAME performance, a face-name associative memory test, is related to higher Aβ deposition in healthy adults with SCD and that the SFN-N may be the most sensitive subtest at detecting Aβ burden.

More specifically, the main finding of this study was that lower performance on the SFN-N (face-name associative memory variable scores, including initial learning, and immediate and delayed recall) were related to higher global Aβ deposition. Moreover, amyloid-positive subjects performed worse on SFN-N than those who were amyloid-negative. This is consistent with results reported by Rentz and coworkers (2011) in an English speaking population with the original FNAME test and using Pittsburgh compound B-positron emission tomography (PiB-PET)^11^.

We used the Spanish version of FNAME (the S-FNAME) to determine whether it was also associated with Aβ burden in subjects with SCD as demonstrated with its original version (the FNAME)^15^. In accordance with previous results^16,17^, the cognitive outcomes most closely related to the face-name associative memory scores of S-FNAME were episodic memory-sensitive NBACE variables (learning, long-term memory and recognition task of the Word List Learning test from WMS-III). These findings reinforce the results obtained in our previous studies using the WMS-III^16^ and with the original validation of FNAME, whose scores were highly correlated with those measured by another recognized episodic memory test, the 6-Trial Selective Reminding Test (SRT)^19^. It reinforces that S-FNAME is a complex associative memory test sensitive to episodic memory.

Concerning the neuroimaging, in contrast to previous studies^15,20^, we used the FBB-PET imaging and found that global, frontal and posterior Aβ deposition values were highly correlated. For this reason, a global Aβ deposition score was used for the analyses. Moreover, both amyloid PiB-PET and FBB-PET Aβ imaging have been demonstrated to have narrow dynamic ranges and similar effect sizes for distinguishing AD from healthy elderly subjects, with a very high correlation between PiB-PET and FBB-PET global Aβ deposition values. The FBB-PET can reliably detect Aβ deposition in the brain without the limitation of the short decay half-life of the radiotracer used in PiB-PET^21^.

In the exploratory Random Forest analysis to explore whether the SFN-N was sensitive to region-specific amyloid load, we found that worse performance on SFN-N were related to higher amyloid burden in bilateral PCC in cognitively healthy subjects with SCD. The Random Forest analysis has allowed identifying the most discriminant specific regional amyloid load. The results of the present study are consistent with the correlation between verbal episodic memory and bilateral PCC metabolism reported previously in subjects with memory complaint^22^. These results may be of special relevance, since the PCC is tightly connected with many other brain regions and it is a central nucleus of the default mode network^23^ and a regulator of cognitive activities^24^. Interestingly, this region has been highly associated with a decrease of gray matter volume in preclinical AD patients^25,26^, showing that subtle brain structural changes occur at the onset of the disease.

Recently, several studies have found a significant relationship between biomarkers and memory tests in cognitively healthy subjects^8,20,27–29^. In addition to Rentz and coworkers study (2011), our findings are also consistent with other studies reporting that lower performance on episodic memory, such as the Rey Auditory Verbal Learning Test^22^ or the 6-Trial SRT^30^, are related to greatest amyloid PiB uptake^12,28^. In our study, except for SFN-N, no relationship was found between SFN-O (face-occupation associative memory variable scores) or the WMS-III (including learning, long-term memory and recognition memory) and global Aβ deposition. This finding is probably due to the fact that SFN-N task is more sensitive than SFN-O and WMS-III. That is, retrieving proper names is more difficult than occupations or common names, possibly because names do not provide semantic information about a subject, whereas other types of words, such as occupations, may be classified according to their semantic categorization and easily associated with different features of their meaning^10,31,32^. Similarly, the association of the face to a name is considered a variable of greater effort in comparison with other memory domains^33,34^. Recent studies support that changes in semantic processing are affected in preclinical AD, mainly involving some aspects of verbal fluency, compared to lexical retrieval-based demands^8^. These findings are consistent with other previous studies^35–37^ describing participants with MCI who routinely show decrements in semantic processing and, in particular, higher declines in category than letter fluency.

In contrast to classical episodic memory tests, such as the 6-Trial SRT and the Word List from the WMS-III, the S-FNAME has been shown to be useful in detecting memory impairment related to amyloid burden in those cognitively healthy subjects with SCD. That is, the S-FNAME may be a useful tool for detecting the first subtle cognitive deficits associated with amyloid burden in preclinical subjects, prior to MCI. Our findings are consistent with those obtained by Rentz and colleagues on the original version of the FNAME and the classical memory test SRT in cognitively healthy individuals.

This study has limitations. First, this is a cross-sectional study, so the results reflect the changes observed in one time point, but should be confirmed in longitudinal follow-up in order to investigate the evolution of these performances and its relationship with increased risk of developing AD dementia. Despite this, the prediction of amyloid positivity, regardless of its prognostic implications, can be considered useful in itself. The new generation of clinical trials targeting preclinical AD is requiring expensive positive amyloid imaging as inclusion criteria, which has led to higher rates of screen failures. Inexpensive and innocuous tools capable of predicting amyloid positivity could be useful for identifying those individuals who should go on to receive amyloid imaging. Finally, our sample size was small, given that we found only 18 subjects who were amyloid positive in a cohort of 200 individuals. Further longitudinal analysis will be needed to determine whether lower SFN-N scores in SCD subjects are related to PCC amyloid burden and an increased risk for future decline and AD dementia.

In conclusion, the results of the present study suggest that S-FNAME may be a useful tool for the detection of AD in its preclinical stage. We found worse performance on SFN-N related to higher levels of cerebral Aβ deposition. Thus, the S-FNAME is a sensitive episodic memory test that shows promise in being able to detect amyloid burden in Spanish speaking subjects without cognitive impairment.

## Methods

### Subjects

All participants were from the Fundació ACE Healthy Brain Initiative (FACEHBI) project^38^ which consists of 200 individuals diagnosed with SCD in the Memory Clinic of Fundació ACE, *Institut Català de Neurociències Aplicades* (Barcelona, Spain)^39^.

All subjects met the following inclusion criteria: they were older than 49 years; literate with at least elementary school (that is, at least 6 years of formal education); with subjective cognitive complaints defined by a score of ≥8 on the Spanish Modified Questionnaire of Memory Failures in Everyday (MFE-30)^40^; with a preserved performance (≥27 scores) on the Spanish version of the Mini-Mental State Examination (MMSE)^41,42^ a strictly normal performance in a comprehensive neuropsychological battery of Fundació ACE (NBACE). This battery has its own normative data for Spanish individuals older than 49 years old^43^ and also, cut-off scores^44^ and includes measures of attention, orientation, information processing speed, verbal learning and memory, language, visuoperception, praxis, and visuospatial and executive functions^43,44^; a Clinical Dementia Rating Score (CDR)^45^ of 0; without relevant depressive/anxiety symptoms (score < 11) on the Spanish version of the Hospital Anxiety and Depression (HAD) Scale^46,47^ or other psychiatric illness; and without severe auditory or visual abnormalities that could affect performance on neuropsychological tests. Exclusion criteria were: evidence of impairment in activities of daily living, presence of psychiatric diagnosis, history of alcoholism or epilepsy, renal or liver failure, and presence of severe auditory or visual abnormalities, including glaucoma and cataracts.

All participants underwent a complete neurological and neuropsychological examination, a set of self-administered questionnaires and a battery of multimodal biomarkers, including the FBB-PET^38^.

### Neuropsychological assessment

As detailed elsewhere^38^, in the FACEHBI project, subjects were administered an extensive neuropsychological protocol which included the NBACE^43,44^ and some additional neuropsychological tests and questionnaires. For the purpose of the present study, all participants were administered the S-FNAME, the classical episodic memory Word List Learning test from the WMS-III and the Vocabulary subtest of the Wechsler Adult Intelligence Scale Third Edition (WAIS-III)^18^ to measure Intelligence Quotient (IQ). As detailed elsewhere^16^ the test begins with an exposure to all 16 faces (face study phase). Subjects were shown 4 faces to a page, 1 face in each quadrant. They were asked to look at each face for a total of 2 seconds until they had seen all 16 faces. To control the time, the examiner used his/her finger to point to each one of the 16 faces for 2 seconds. The subject had to read the name below and try to learn each face-name pair. The same procedure was repeated with the 16 face-occupation pairs.

In the *Initial study of face–name pairs (SFN–N)*, subjects were then presented the same 16 faces with names underneath and were asked to study the name that goes with the face. Subjects were given only one exposure to learn all 16 SFN–N pairs. In the *Initial cued recall of face–name pairs*, the subjects were then shown the face and were asked to recall the name that goes with the face. The correct number of SFN–N pairs was recorded as an initial learning score for names (S-ILN). In the *Initial study of face–occupation pairs (SFN–O)*, subjects were shown the same faces, but this time with occupations underneath. The SFN–O pairs were presented in the same manner as the SFN–N pairs until all 16 SFN–O pairs were studied. *Initial cued recall of face–occupation pairs:* Subjects were again shown the face and were asked to recall the occupation that goes with the face. Correct recall of SFN–O pairs was tabulated as initial learning of occupations (S-ILO). In the *Immediate cued recall*, subjects were shown the face and were asked to recall the name (S-CRN) and occupation (S-CRO) that was associated with the face. In the *30-minute delayed cued recall*, subjects were again presented the face and were asked to recall the name (S-CRN30) and occupation (S-CRO30) associated with the face. Score for each S-FNAME subscale (S-ILN, S-ILO, S-CRN, S-CRO, S-CRN30 and S-CRO30) ranged from 0 to 16, subtotal scores for names (SFN-N = S-ILN + S-CRN + S-CRN30) and occupations (SFN-O = S-ILO + S-CRO + S-CRO30) were out of 48, and total score for S-FNAME (S-ILN + S-ILO + S-CRN + S-CRO + S-CRN30 + S-CRO30) was out of 96. Taking into account that S-CRN30 and S-CRO30 subtests might be administered 30 minutes after the initial subtests, the S-FNAME takes between 35 and 40 minutes.

### Development of Composite Factor Scores

All variables of the S-FNAME and the Word List Learning test from the WMS-III^18^ were analyzed in a Principal Component Analysis to obtain composite factor scores of episodic memory.

### Statistical analysis

Statistical analysis of the clinical variables was performed using SPSS (version 20.0; SPSS Inc., Chicago, IL). All data were examined for normality, skew, and restriction of range. T-tests were used to compare age and education between men and women. Pearson’s correlation analyses were carried out to explore the relationship between all Word List Learning test from the WMS-III and S-FNAME subtests and total scores; between Standard Uptake Value Ratio (SUVR) of Frontal, PPCLP and Global cortices; and between the 3 episodic memory composites and the Global SUVR cortex data.

As mentioned above, to determine whether the episodic memory variables of S-FNAME and Word List Learning test from the WMS-III were grouped in face-name, face-occupation and a classical memory test components, similar to Rentz *et al*.^15^ study, a Principal Component Analysis (PCA) was carried out. The variables introduced in the analysis were the following: S-CRN30, S-CRN, S-ILN, S-CRO, S-CRO30, S-ILO and WMS-III variables (verbal learning [first + second + third + fourth trial scores], delayed recall [free recall of words] and recognition [cued recall of words]).

To determine whether the SFN-N was sensitive to region-specific amyloid load, a total of 19 bilateral cerebral regions were included in the analysis. According to the exploratory aim of the present analysis, and in order to avoid multicollinearity between predictors and/or overfitting of the resulting model, it was needed to select the most relevant predictors in order to be later analyzed in a standard multiple regression model. Random Decision Forest analysis was implemented^48^. Random forest is an ensemble learning strategy usually implemented to identify the most discriminant variables. This approach combines two random principles: Resampling at random the subjects of the sample, and selecting sets of predictors, again at random, in each execution. These random strategies, convergent in a single analysis, are executed hundreds or thousands of times, providing thus hundreds or thousands of trees. These trees are later combined between them obtaining a more consistent and reproductively result. One interesting characteristic of the technique in an exploratory approach, as in the present study, is its capacity to provide a ranking of the best predictors included in each single model, counting the times that each predictor was located at the first node, the most discriminant one, among all the obtained trees. This strategy provides a high discriminative result, comparable to a classical Tree Decision technique, but avoiding overfitting. In this study, 10000 trees were executed including the 19 predictors and taking the three composites, in separate analyses, as variables to be discriminated. Gain ratio rule was applied as a splitting criterion^49^. The Knime Analytics Platform v3^50^ was used to execute Random Forests. There are no standard criteria to decide how many predictors have to be selected between the most discriminant one to be analyzed later in a standard inferential statistical procedure. In this study, the first 5 predictors were selected for each composite. These predictors were later analyzed together in a multivariate lineal regression, using a stepwise selection procedure to identify the final significant variables. A test for multicollinearity was executed in each regression analysis.

To compare performance on S-FNAME between subjects with Aβ positivity and Aβ negativity (that is, FBB-PET positive and FBB-PET negative groups), t-test analyses were carried out. Multiple regression analyses were performed relating Global cortical Aβ deposition as a continuous variable (Global SUVR) and memory performance using the 3-factor scores (SFN-N, SFN-O and WMS-III Composites), corrected for age and Vocabulary WAIS-III IQ score. Moreover, to describe the ability to discriminate between both groups, sensitivity and specificity values were calculated for the 3-factor scores using Logistic Regression analyses. Corrections for multiple comparisons were done using Bonferroni correction. Significance was defined at the *p* < 0.05 level and all hypotheses were tested directionally at a 95% confidence level.

### FBB-PET neuroimaging processing

FBB-PET images were acquired for all subjects of the study in a 90 days window after the baseline visit. The MRI T1-3D of 1 × 1 × 1 mm voxel size was acquired with a 1.5 T Siemens^©^ Magneton Aera. FBB-PET scans were acquired in a Siemens^©^ Biograph molecular-CT machine. Four FBB-PET scans of 5 minutes were acquired after 90 minutes post injection of 300 Mbq of Florbetaben(18 F) radio tracer (NeuraCeq^©^). FBB-PET scans were processed with FSL 5.0 suite. The FBB-PET images were coregistered onto structural MR images. Prior to the alignment between PET and MRI data, MRI segmentation was carried out with Freesurfer 5.3, identifying regions from the Desikan-Killiany Atlas^51^. SUVR was determined as the mean value of the cortical regions segmented on MRI and normalized by the cerebellum. The SUVR were calculated in aggregate cortical regions-of-interest (ROIs) for the frontal association cortex constituted by inferior (opercularis, triangularis, orbitalis), superior and middle gyri aside from the supplementary motor area; the precuneus, posterior cingulate and lateral parietal cortices (PPCLP); and Global cortex. Besides aggregates, the SUVR from segmented structures were also explored as targets for the current study. A cut-off of SUVR = 1.45 was selected as amyloid positive criterion, that is, to classify subjects in FBB-PET positive and FBB-PET negative groups^52^.

### Ethical standards

Prior to the evaluation a written informed consent was obtained from all participants. The FACEHBI protocol received approval from the ethics committee of the Hospital Clinic i Provincial (Barcelona, Spain) (EUdraCT number 2014-000798-38). The referral center ethics committee approved the patient recruitment and collection protocols were in accordance with ethical standards according to WMA Declaration of Helsinki-Ethical Principles for Medical Research Involving Human Subjects.
